# Prediction of methane per unit of dry matter intake in growing and finishing cattle from the ratio of dietary concentrations of starch to neutral detergent fiber alone or in combination with dietary concentration of ether extract

**DOI:** 10.1093/jas/skac243

**Published:** 2022-07-27

**Authors:** Michael L Galyean, Kristin E Hales

**Affiliations:** Department of Veterinary Science, Texas Tech University, Lubbock, TX 79409, USA; Department of Animal and Food Science, Texas Tech University, Lubbock, TX 79409, USA

**Keywords:** beef cattle, ether extract, methane prediction, starch:neutral detergent fiber ratio

## Abstract

Previous research demonstrated that a fixed value of 0.2433 (SE = 0.0134) Mcal of CH_4_/kg of dry matter intake (**DMI**) could be used to predict CH_4_ production with accuracy and precision on par with similar equations in the literature. Slope bias was substantially less for the fixed-coefficient equation than noted for the other DMI- or gross energy intake (**GEI**)-based equations, but mean bias was substantially greater, presumably reflecting the failure of the fixed-coefficient approach to account for dietary factors that affect CH_4_ production. In this article, we report on the use of the dietary ratio of concentrations of starch to neutral detergent fiber (**NDF**) and dietary ether extract (**EE**) concentration to improve the accuracy and precision of the fixed-coefficient equation. The same development data set used to create the fixed-coefficient equation was used in the present study, which included 134 treatment means from 34 respiration calorimetry studies. Based on stepwise regression with dietary NDF, starch, crude protein, EE, and the starch:NDF ratio as possible dependent variables, the starch:NDF ratio and EE were the only dietary variables selected (*P* ≤ 0.15). The study-adjusted relationship with the starch:NDF ratio (*r*^2^ = 0.673; root mean square error [**RMSE**] = 0.0327) was: Mcal of CH_4_/kg of DMI = 0.2883 − 0.03474 × starch:NDF; whereas the relationship with a model that included both starch:NDF ratio and dietary EE (*r*^2^ = 0.738; RMSE = 0.0315) was: Mcal of CH_4_/kg of DMI = 0.3227 − 0.0334 × starch:NDF − 0.00868 × % EE. A previously published independent data set with 129 treatment means from 30 respiration calorimetry studies was used to evaluate these two equations, along with two additional equations in which g/d of CH_4_ was predicted directly from DMI, starch:NDF ratio, and/or dietary EE. The two Mcal of CH_4_/kg of DMI equations had superior fit statistics to the previously published 0.2433 Mcal of CH_4_/kg of DMI equation, with a substantial decrease in mean bias and improved concordance correlation coefficients. Moreover, the Mcal of CH_4_/kg of DMI equations resulted in improved fit relative to direct prediction of g/d of CH_4_ from DMI, the starch:NDF ratio, and % EE. Based on these results, further evaluation of the dietary ratio of starch-to-NDF concentrations and EE concentration to predict methane production per unit DMI in beef cattle is warranted.

## Teaser

Equations to predict the megacalories of methane per unit of dry matter intake from the ratio of dietary starch to neutral detergent fiber concentrations and dietary ether extract concentration yielded better fits to experimental data than equations that directly predicted methane from the same variables in combination with dry matter intake. Further research is needed to evaluate these equations in other independent data sets.

## Introduction

Given environmental concerns about the contributions of ruminant production systems to greenhouse gas emissions, development of accurate and precise equations to predict CH_4_ is an important area of research. Using a data set derived from energy balance studies with growing and finishing beef and dairy cattle, Hales et al. (2022) demonstrated that multiplication of DMI by a fixed value of 0.2433 Mcal of CH_4_/kg of DMI could be used to predict daily CH_4_ production. When evaluated with an independent data set, the fixed-value equation gave similar or greater *r*^2^, root mean squared prediction error (**RMSPE**), and concordance correlation coefficients (**CCC**) to extant equations based on DMI or GEI; however, the mean bias of the fixed-coefficient equation was approximately 34% of the RMSPE, a value 10 times greater than the other equations that were compared. Adjusting the fixed-coefficient equation for the potential effects of level of intake above maintenance did not markedly improve fit statistics and had no effect on mean bias. The authors suggested that because the predicted values were consistently less than observed values across the range of the data, the fixed-coefficient needed adjustment, presumably for differences in diet composition. Specifically, differences in dietary concentrations of neutral detergent fiber (**NDF**), starch, and ether extract (**EE**) between the top and bottom 50% of values in their development data set were noted as potential discriminating factors.

In the present report, we extend the work of Hales et al. (2022) and demonstrate the effectiveness of adjusting the fixed coefficient for the effects of the ratio of dietary starch to NDF concentrations alone or in combination with dietary EE concentration. Newly developed equations were then tested with the same independent evaluation data set used by Hales et al. (2022).

## Materials and Methods

Data used in this article were generated from published literature; thus, no live animals were used by the authors, and Institutional Animal Care and Use Approval was not necessary.

### Data sets and statistical methods

The development and evaluation data sets described by Hales et al. (2022) were used. Briefly, the development data set included 134 treatment means from 34 respiration calorimetry studies conducted with growing finishing beef cattle and dairy steers and heifers. The evaluation data set consisted of 129 treatment means from 30 respiration calorimetry studies conducted with beef and dairy steers and heifers. The complete development and evaluation data sets are available in spreadsheet format as supplementary material in Hales et al. (2022).

#### Adjusting the fixed-coefficient equation

Initial analyses to evaluate the possibility of adjustments to the fixed coefficient for prediction of Mcal of CH_4_/kg of DMI from Hales et al. (2022) were conducted using PROC STEPWISE of SAS (SAS Inst. Inc., Cary, NC; version 9.4). Dietary composition values in the development data set (NDF, starch, crude protein, and EE) were considered as potential independent variables for model selection, with the *P*-value for entry into the model set at 0.15. Given that the ratio of starch to NDF has been shown to have a predictive value for CH_4_ (MJ/d; Ellis et al., 2009), it also was included in the list of possible independent variables. Because several diets in the development data set did not include measurable quantities of starch, to avoid division by zero, starch must be the numerator of the ratio.

Mixed model methods described by Littell et al. (2006) were subsequently used to evaluate the relationship between dietary Mcal of CH_4_/kg DMI and the selected dietary variables in the development data set. In addition, the g/d of CH_4_ produced was evaluated using DMI and the same dietary variables included in the Mcal of CH_4_/kg of DMI equations. Study was included in the models as a random effect to account for variation from different intercepts in the published studies. The covariance structure used for these analyses was unstructured, and the estimation method was restricted maximum likelihood. Random slope effects were considered, but for all models, the Akaike information criterion was increased when random slopes were added; thus, random slope effects were not included in the final models. Study-adjusted data were created for each data point from the linear models (Galyean and Tedeschi, 2014). The coefficient of determination (*r*^2^) and root mean square error (**RMSE**) were determined for the model using the study-adjusted values and PROC MIXED and PROC REG of SAS (SAS Inst. Inc., Cary, NC; version 9.3).

The independent evaluation data set of Hales et al. (2022) was used to evaluate the ability of the new equations to predict CH_4_ by regressing observed CH_4_ on the predicted CH_4_ for each equation. No adjustments were made for the source of the data (study) in the independent evaluation data set. For these analyses, daily methane production expressed in Mcal/d was converted to g/d using conversion factors of 9.45 kcal/L and 0.716 g/L for methane. In addition to the coefficient of determination and root mean square prediction error (**RMSPE**) statistics, the CCC was computed as described by Lin (1989), and the mean squared prediction error (**MSPE**) was decomposed by determining the mean, slope, and error biases and expressing these values as a percentage of the MSPE (Tedeschi, 2006).

## Results and Discussion

### Predicting megacalories of CH_4_ per unit of dry matter intake

Using the same development data set employed in the present study, Hales et al. (2022) reported that by fitting a model with no slope but an adjustment for random intercepts associated with studies, the energy lost as CH_4_ (Mcal/kg of DMI) was 0.2433 Mcal/kg of DMI (SE = 0.0134; 95% confidence limits = 0.216 and 0.271). Multiplication of the 0.2433 Mcal/kg value by DMI yield an estimate of Mcal of daily CH_4_ production. Hales et al. (2022) further evaluated the relationship in the development data set between multiples of maintenance intake and daily energy lost as methane (Mcal/kg of DMI), with a resulting equation (adjusted for slope and intercept effects) as follows: CH_4_, Mcal/kg DMI = 0.3344 − 0.05639 × multiple of maintenance (*r*^2^ = 0.536, RMSE = 0.0245).

Hales et al. (2022) compared these two equations with other equations in the literature that used either DMI or GEI by evaluating the relationships between observed and predicted CH_4_ production in the independent data set described previously. Based on fit statistics reported by Hales et al. (2022), the two equations gave similar or greater *r*^2^, RMSPE, and CCC to other equations, as well as lower slope bias, but the mean bias of both the fixed-coefficient equation and the fixed coefficient adjusted for multiples of maintenance intake was approximately 34% of the RMSPE, which was 10 times greater than the other equations that were compared. These results led Hales et al. (2022) to suggest that adjustment of the fixed-coefficient equation for dietary or animal factors that might decrease mean bias and improve the accuracy and precision of predicting CH_4_ production should be evaluated. In particular, Hales et al. (2022) noted differences in dietary concentrations of NDF, starch, and EE among observations in the development data set and suggested that parsing out groups of data with similar dietary characteristics might be a way to refine the fixed-coefficient approach.

Our current analyses represent an effort to adjust the fixed-coefficient equation of Hales et al. (2022), specifically to address the potential effects related to dietary components. From the stepwise regression analysis with dietary NDF, starch, crude protein, EE, and the starch:NDF ratio as possible dependent variables, only the starch:NDF ratio and EE met the *P*-value threshold (≤0.15) for entry into the model. The starch:NDF ratio was the first variable selected in the stepwise analysis, with an *r*^2^ of 0.503, whereas adding EE to the model increased the *r*^2^ to 0.542. Because starch:NDF accounted for most of the variation in Mcal of CH_4_/kg of DMI, two models, one with starch:NDF only and another with starch:NDF and EE were evaluated in the mixed model analyses to account for random intercepts of studies, resulting in the following two equations:


$$\begin{array}{ll} Mcal\;of\;CH_{4}/kg\;of\;DMI\;= & \;0.2883-0.03474\;\\ & \times \;starch\;:\;NDF; \end{array}$$ [1]

where *r*^2^ = 0.673; RMSE = 0.0327; 95% confidence intervals: intercept = 0.2807, 0.2959 and starch:NDF slope = –0.0389, –0.0306.


$$\begin{array}{ll} & Mcal\;of\;CH_{4}/kg\;of\;DMI\;=\;0.3227-0.0334\;\\ & \times \;starch\;:\;NDF-0.00868\;\times \;\%\;EE; \end{array}$$ [2]

where *r*^2^ = 0.738; RMSE = 0.0315; 95% confidence intervals: intercept = 0.3104, 0.3350, starch:NDF slope = –0.0376, –0.0292, and EE slope = –0.0115, –0.0059.

The resulting value for Mcal of CH_4_/kg of DMI from equations 1 and 2 is multiplied by DMI to determine Mcal of CH_4_ produced and then converted to g/d. An alternative to predicting Mcal of CH_4_/kg of DMI and then converting that value to g/d of CH_4_ is to directly predict g/d of CH_4_ from DMI, starch:NDF, and EE. Initial stepwise regression analysis indicated that when given DMI (kg/d), starch, NDF, starch:NDF, crude protein, and EE as potential independent variables, only DMI, starch:NDF, and EE entered the model at the *P* ≤ 0.15 level. The subsequent mixed-model analyses adjusting for random effects of study resulted in the following two equations:


$$\begin{array}{l} CH_{4},\;g/d\;=\;32.2935\;+\;15.7637\;\\ \times \;DMI-15.7420\;\times \;starch\;:\;NDF; \end{array}$$ [3]

where *r*^2^ = 0.812, RMSE = 14.1297; 95% confidence intervals: intercept = 23.7361, 40.8509, DMI slope = 14.2939, 17.2335, and starch:NDF slope = –17.6259, –13.8581.


$$\begin{array}{ll} CH_{4},\;g/d\;= & \;48.3465\;+\;15.3643\;\times \;DMI\;-15.1229\;\\ & \times \;starch\;:\;NDF-\;3.4998\;\times \;\%\;EE; \end{array}$$ [4]

where *r*^2^ = 0.836, RMSE = 13.7426; 95% confidence intervals: intercept = 34.4663, 58.2267, DMI slope = 13.9232, 16.8054, starch:NDF slope = –17.0409, –13.2049, and EE slope = –4.7247, –2.2750.

**Figure 1.**
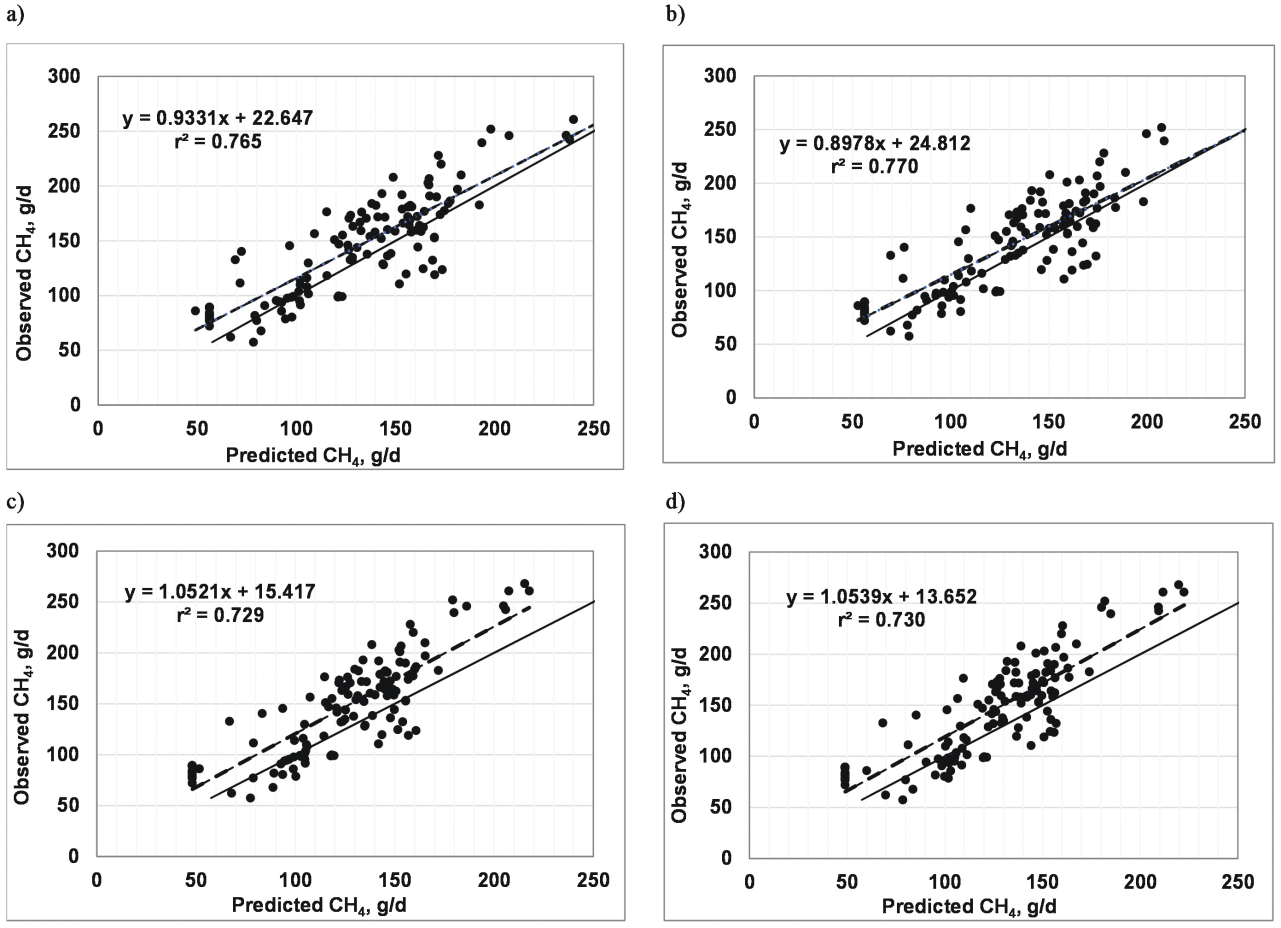
Plot of observed vs. predicted methane (g/d) using equation 1 (a), equation 2 (b), equation 3 (c), and equation 4 (d) developed in this study. The solid line indicates *y* = *x*, and the dashed line is the regression observed on the predicted values.

Using the evaluation data set of Hales et al. (2022), the regression of observed g/d of CH_4_ on CH_4_ predicted from equations 1 through 4 resulted in the fit statistics shown in Table 1. The relationships are shown graphically in Figure 1. Equations 1 and 2 from the current analyses had superior fit statistics (greater *r*^2^, smaller RMSPE, and higher CCC) compared with the fixed-coefficient equation and the fixed-coefficient equation adjusted for multiples of maintenance intake, as well as the other published equations evaluated by Hales et al. (2022; see Table 2 in that publication). Moreover, equations 1 and 2 had mean biases that were from 25 to more than 45% less than the two equations developed by Hales et al. (2022), indicating that adjusting the fixed-coefficient approach for differences among diets in the starch:NDF ratio and EE concentrations was an effective means of improving the accuracy and precision of CH_4_ predictions. Equations 3 and 4, in which g/d of CH_4_ is predicted directly, had noticeably inferior fit statistics compared with equations 1 and 2, particularly in terms of the RMSPE, mean bias, and CCC. The superior fit of equations 1 and 2 vs. Equations 3 and 4 presumably reflects multiplication of the predicted Mcal/kg of DMI by the known DMI vs. multiplication of the known DMI by a slope coefficient with its associated error when g/d of CH_4_ is predicted directly.

**Table 1.** Equation performance statistics for the four equations developed in the current study to predict daily methane emission

Item	*r* ^2^	RMSPE^a^, g/d	RMSPE, % of mean	% of RMSPE			CCC^b^
				Mean bias	Slope bias	Error bias	
Equation 1	0.765	27.7	19.2	25.5	1.2	73.3	0.84
Equation 2	0.770	26.5	18.4	18.1	3.4	78.5	0.85
Equation 3	0.729	33.6	23.3	42.2	0.4	57.5	0.75
Equation 4	0.730	32.6	22.6	38.9	0.4	60.7	0.76

RMSPE = root mean square prediction error.

CCC = concordance correlation coefficient.

Dry matter intake is an important driver of CH_4_ production in ruminants (Congio et al., 2022). Thus, including DMI in prediction equations or evaluation of CH_4_ production per unit DMI as was done in the present study are typical approaches used in developing empirical regression equations to predict CH_4_ production. The starch:NDF ratio seems to provide a convenient means of describing the effects of dietary components that are known to affect CH_4_ production. In addition, dietary EE has been shown to be a potentially important predictor of CH_4_ (van Lingen et al., 2019). As noted previously, Ellis et al. (2009) reported that the starch:NDF ratio and DMI were potentially effective predictors of CH_4_ production in beef cattle. These authors used a much larger database than our development data set, and one in which CH_4_ was measured by a variety of methods in addition to chambers and head boxes. Like ours, their equation was adjusted for random effects of data source. Nonetheless, the agreement between the equation by Ellis et al. (2009) that included the starch:NDF ratio and our comparable equation (equation 3) is striking. If the equation by Ellis et al. (2009) is converted to g/d of CH_4_ rather than MJ/d as originally reported, the converted equation is as follows: CH_4_, g/d = 48.53 + 14.23 × DMI − 20.64 × starch:NDF. Based on the standard errors presented by Ellis et al. (2009), the 95% confidence intervals for the intercept and the slope for DMI overlap with those of equation 3, whereas the confidence intervals for the starch:NDF ratio slope do not. When the starch:NDF ratio equation by Ellis et al. (2009) was used to predict g/d of CH_4_ in our evaluation data set, the correlation between those values and CH_4_ predicted from equation 3 was 0.99, an indication of the strong agreement between the equations.

Further refinement of the starch:NDF ratio approach is possible. For example, the degradability of starch in the rumen is markedly affected by grain processing (Owens et al. 1986). Adjusting the starch:NDF ratio for degradability in the rumen (i.e., degree of processing or degree of starch gelatinization) might improve predictability. In addition, the source of NDF could have important effects on methane production. The NDF from roughage sources such as hays and fibrous crop residues is compositionally and structurally different from NDF in grains and grain byproducts, resulting in less physical effectiveness of non-roughage fiber sources (Armentano and Pereira, 1997). Perhaps defining the roughage or forage NDF component of the total NDF would improve CH_4_ predictions.

Present data suggest that using dietary ratio of starch to NDF concentrations alone or in combination with dietary EE concentration is effective in predicting Mcal of CH_4_/kg of DMI. Important improvements in fit statistics were noted using this approach compared with a fixed-coefficient approach reported by Hales et al. (2022), and the potential importance of these dietary variables is supported by previous research. Additional development of this approach and evaluation with other independent data sets is warranted.

## Abbreviations

CCC: concordance correlation coefficient
DMI: dry matter intake
EE: ether extract
GEI: gross energy intake
MSPE: mean squared prediction error
NDF: neutral detergent fiber
RMSE: root mean square error
RMSPE: root mean square prediction error
